# MAPK Signaling Pathway Regulates p27 Phosphorylation at Threonin 187 as Part of the Mechanism Triggered by Early-Weaning to Induce Cell Proliferation in Rat Gastric Mucosa

**DOI:** 10.1371/journal.pone.0066651

**Published:** 2013-06-07

**Authors:** Luciana H. Osaki, Patricia Gama

**Affiliations:** Department of Cell and Developmental Biology, Institute of Biomedical Sciences, University of São Paulo, São Paulo, Brazil; Cincinnati Children's Hospital Medical Center, United States of America

## Abstract

During rat postnatal development, gastric cell proliferation and differentiation depend on many elements, which include dietary pattern, hormones, growth factors and their signaling pathways. Among them, EGFR activity is increased through MAPK and Src cascades in response to early weaning that represents the abrupt transition from milk to solid food. We herein investigated the direct involvement of ERK pathway in the control of cell cycle progression during early weaning, and studied the specific role of p27. At 15 days, Wistar rats were separated from dams, fed with powdered chow and daily injected with PD98059 (MEK inhibitor, 300 µg/kg) or 0.5% DMSO (control). By using HE staining and immunohistochemistry for PCNA, we respectively detected mitotic (MI) and proliferative (PI) indices in 18-day-old pups, and observed that both were reduced by PD98059. As cell cycle-related proteins (cyclin E, CDK2, cyclin D1, CDK4, p21 and p27) are involved in proliferative regulation, we compared samples obtained at 17 days in the morning (17 d) and evening (17.5 d). We found that they were not altered after ERK inhibition, but cyclin D1, p21 and p27 levels changed throughout the day in the control group. As p27 activity depends on its integrity, we studied p27 phosphorylation (threonin 187), and observed that ERK inhibition reduced this process. We suggest that MAPK pathway interferes in the regulation of p27 function in the gastric mucosa during early weaning, possibly by controlling its degradation, and altogether this mechanism might contribute to the increase of epithelial proliferation at this condition.

## Introduction

Cell cycle progression is regulated by several proteins, and among them cyclins and cyclin-dependent kinases (CKDs) play important roles. Mammalian cells express different cyclins and CDKs, which associate with each other to constitute different complexes [1]–[4]. Besides cyclin binding, CDK activity also comprises the association with cyclin-dependent kinase inhibitory proteins (CKI) and phosphorylation events. There are two major families of CKIs: p15, p16, p18 and p19 that belong to the INK4 group, and p21, p27 and p57 that are members of the Kip/Cip family. Whereas INK4 proteins specifically inhibit CDK4 and CDK6, Kip/Cip peptides are capable of binding a broad range of CDKs. Although p27^Kip1^ (p27) was first described as an inhibitor of cyclin E-CDK2 [5], it also promotes the assembly and nuclear import of cyclin D-CDK4/6 [6], [7], which contribute to cell cycle progression. The regulation of p27 function occurs at transcriptional, translational and post-translational levels,[8] which include the degradation step through ubiquitylation followed by proteasomal activity [9]. In order to be targeted to destruction, cyclin E-CDK2 complex phosphorylates p27 at threonin 187 (T187) [10], and phospho-p27 is then ubiquitylated [11] by the F-box protein SKP2 [12], and so, p27 is degraded through the ubiquitin-proteasome system (UPS).

Many factors can generate pro- and anti-mitogenic signals capable of interfering in cell cycle progression. In the gastric mucosa, cell proliferation is controlled by an interaction of endogenous and exogenous elements, such as hormones, growth factors, diet, luminal microbes and genetic program [13], [14]. Milk-borne molecules and feeding pattern have emerged as important keys in the regulation of different cellular processes in the gastric epithelium during postnatal development [15]–[19]. Interestingly, when suckling is interrupted by early weaning, several effects are observed in the rat gastric mucosa, such as the rapid differentiation of mucous neck cells [15], the augment of pepsinogen and ornithine-decarboxylase activities [16], and increased cell proliferation [17]. Moreover, we have previously shown that early weaning also influences the expression of growth factors and activation of signaling pathways. Accordingly, the abrupt change from milk to solid food modifies TGFβ3 localization [18], increases TGFα and EGFR protein levels [15], and stimulates ERK and Src phosphorylation in the gastric mucosa [20]. As mentioned before, early weaning stimulates gastric cell division [17], and such effect is reverted after the inhibition of EGFR phosphorylation [20], indicating that this receptor is part of the response. However, the mechanisms triggered by EGFR to locally stimulate gastric cell proliferation are still unknown.

Many signaling pathways can be activated to accelerate growth during early weaning [21], and we have demonstrated that the phosphorylation of ERK1/2 and Src is augmented in rats submitted to this feeding pattern [20]. It is known that ERK1/2 pathway is mainly associated to proliferative stimulus, but other functions are also attributed to these proteins, including the modulation of cell death, migration and differentiation, and such variability depends on the many substrates that can be activated by ERK1/2 [22]. As for cell cycle control, MAPK phosphorylates p27 *in vitro*, and so, it decreases the inhibitory function of this CKI, since it becomes unable to bind CDK2 [23]. Additionally, high levels of ERK1/2 induce cyclin D expression, also demonstrating the pro-proliferative role of MAPK [24].

Because ERK activation is increased in the gastric epithelium of early-weaned animals in parallel to stimulated cell proliferation, and MAPK signaling pathway is tightly related to cell cycle control, we investigated whether this cascade might be essential for early weaning effects. Firstly, we showed that the inhibition of MEK activation decreased gastric cell proliferation in early-weaned rats. Then, we investigated the mechanisms that could be involved in this process by analyzing cell cycle-related proteins, and we found that the levels of p21, p27, cyclin D, cyclin E, CDK2 and CDK4 were not altered after MEK inhibition. However, the phosphorylation of p27 at threonin 187 was low in PD98059-treated animals, suggesting that the reduction of ERK1/2 signaling decreased the targeting of this CKI to degradation. Taken together, our results demonstrated that MAPK pathway is directly involved in the proliferative stimulus triggered by early weaning in the rat gastric epithelium, probably through the regulation of p27 phosphorylation and degradation.

## Results

### PD98059 reduces ERK activation in the rat gastric epithelium during early weaning

**Figure 1:**
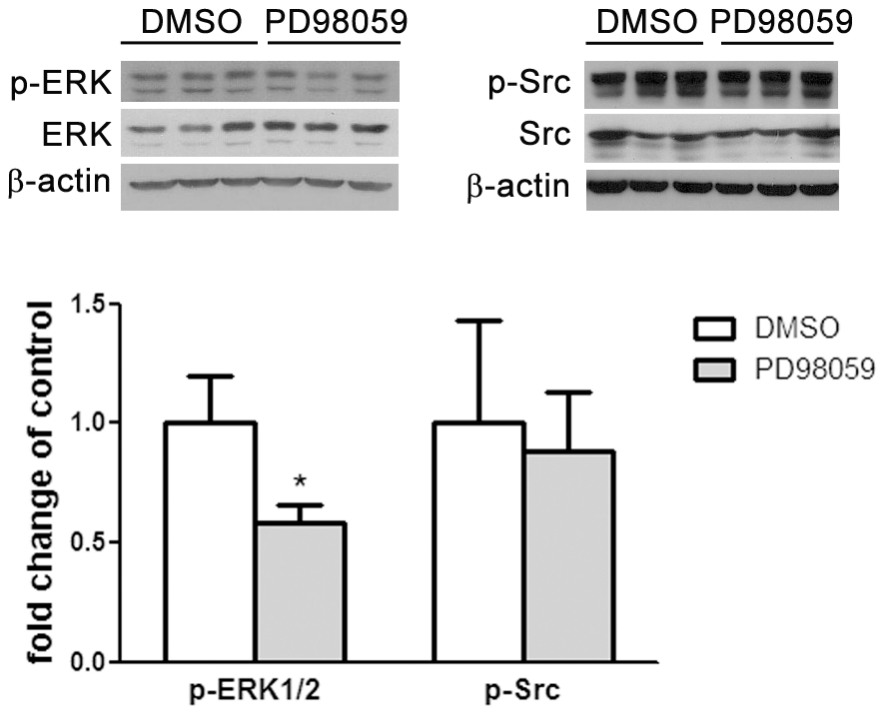
PD98059 inhibits ERK1/2 phosphorylation in 17-day-old rats submitted to early weaning. Animals were treated with 0.5% DMSO (control) or PD98059 at 300 µg/kg. Immunoblots and the respective densitometries are shown for p-ERK1/2, ERK1, p-Src and Src. Thirty µg of total protein were applied to each lane and each band is representative of one animal. β-actin was used as loading control. Relative densitometries as ratio of p-ERK/ERK1 and p-Src/Src are shown as fold change of control group and are presented by bars as means±SD (n = 3/group). *p<0.05 when comparing PD98059-treated group to control. Assays were conducted in duplicates.

Early weaning stimulates ERK and Src signaling pathways in the rat gastric epithelium [20] and we investigated the role of ERK in gastric cell proliferation by treating early-weaned pups with PD98059 to block ERK phosphorylation through inhibition of MEK activity. Early weaning was performed by separating pups from dams at 15 days and placing them in plastic cages with water and hydrated powdered chow offered *ad libitum*. PD98059 at 300 µg/kg was daily administered to early-weaned pups and control groups received DMSO injections. First, we evaluated the efficiency and specificity of the dose used for PD98059 treatment using Western blot analysis as previously described [20]. At 17 days, animals from both PD98059-treated and control groups were submitted to euthanasia and samples were obtained by scraping the mucosa from the corpus region of the stomach. After extraction, 30 µg of protein were separated in a 5–20% gradient SDS-PAGE, transferred into nitrocellulose membrane and submitted to immunoblot for p-ERK, ERK, p-Src, Src and β–actin. We found that PD98059 decreased the phosphorylation of ERK when compared to the control group at 17 days (p<0.05) (**Figure 1**), indicating that the dose was efficient to reduce ERK activation. We also observed that p-Src levels did not change after PD98059 administration, and remained as high as during early weaning [20] (**Figure 1**).

### ERK is involved in early weaning-induced cell proliferation

**Figure 2:**
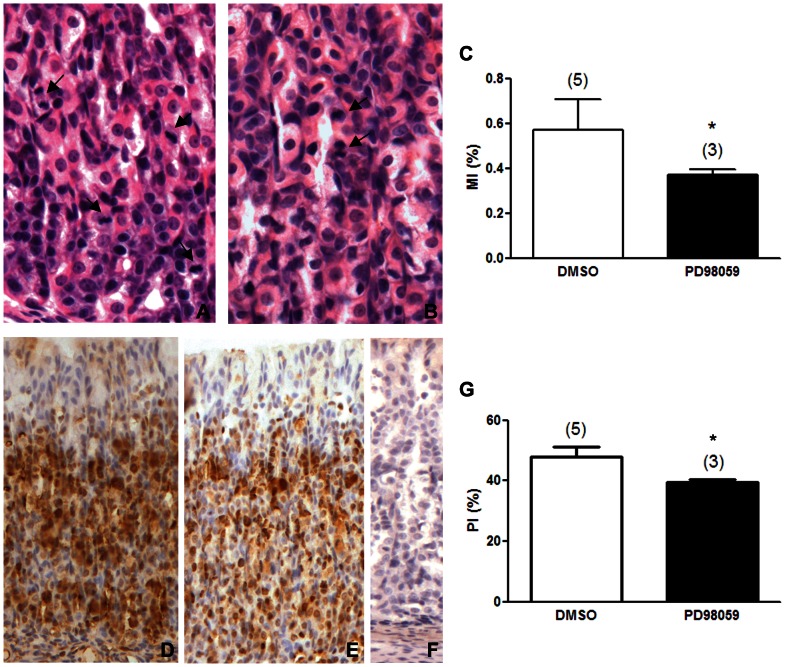
Inhibition of MAPK phosphorylation impairs the proliferation in the gastric epithelium in early-weaned rats. (A and B) Hematoxylin & Eosin stained sections of the gastric mucosa of 18-day-old early-weaned rats. Arrows indicate mitotic cells. Original magnification: 40×. (D and E) Immunohistochemistry for PCNA developed with DAB and counterstained with Mayer's Hematoxylin. (F) Negative control. Original magnification: 20×. (C) Mitotic Index (MI) and (G) Proliferative Index (PI) in 18-day-old early weaned rats treated with 0.5% DMSO (control) or PD98059 at 300 µg/kg. Indices are represented by bars as means±SD. MIs and PIs were obtained after counting 2,500 epithelial cells/animal, and they represent the number of mitotic or PCNA-labeled cells/total number of epithelial cells. (n) number of animals in each group. Results were analyzed statistically by Student *t* test. *p<0.05 when compared to the control group.

We studied the role of ERK activation in gastric epithelium cell proliferation during early weaning by using mitotic (MI) (**Figure 2A**–**C**) and proliferative indices (PI) (**Figure 2D**–**G**) which were determined in 18-day-old animals from control (**Figure 2A, D**) or PD98059-treated groups (**Figure 2B,E**). We counted mitotic and interphasic cells in HE stained sections (**Figure 2A,B**) and detected PCNA through immunohistochemistry (**Figure 2D**–**F**) to respectively estimate the number of dividing cells (MI) (**Figure 2C**), and the number of labeled and non labeled cells (PI) (**Figure 2G**). PD98059 significantly decreased the MI (**Figure 2C**) in the gastric epithelium of early-weaned animals when compared to control group treated with DMSO (vehicle) (p<0.05). Similarly, the PI index was also reduced by PD98059 treatment when compared to control animals (p<0.05) (**Figure 2G**), confirming that ERK was activated to induce cell proliferation during early weaning.

### ERK signaling and cell cycle-related proteins

**Figure 3:**
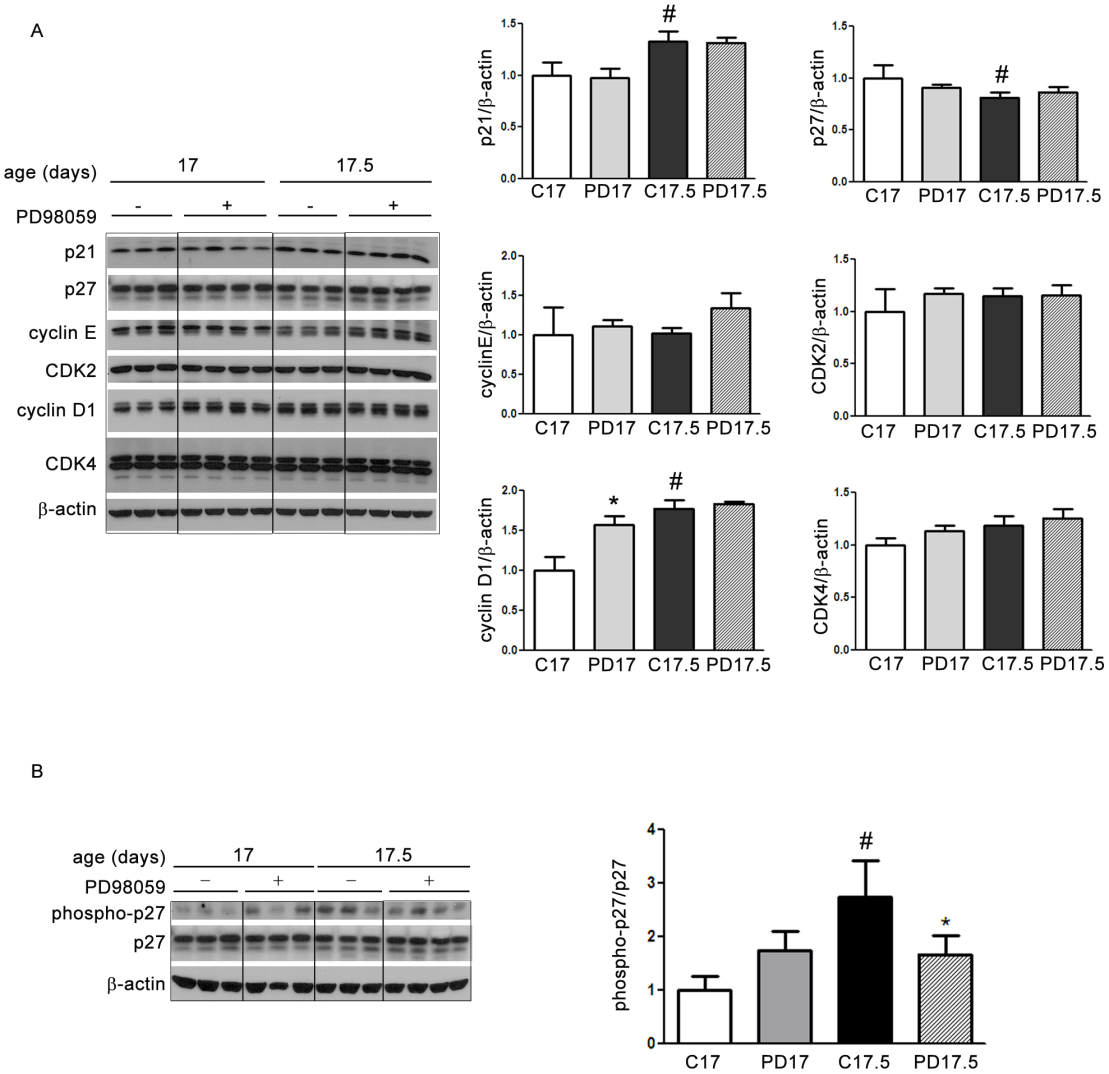
ERK inhibition and cell cycle-related proteins in the gastric mucosa of 17-day-old early-weaned rats. Animals were treated daily with 0.5% DMSO (control) or PD98059 at 300 µg/kg and samples were collected in the morning (17 d) and evening (17.5 d). Immunoblots and the respective densitometries are shown for (A) p21, p27, cyclin E, CDK2, cyclin D1, CDK4 and (B) phospho-p27. Thirty µg of total protein were loaded into each lane and each band is representative of one animal. β-actin was used as loading control. Relative densitometry as ratio of β-actin (A) or total p27 (B) is shown as fold change of control group (17 d) and is presented by bars as means±SD (n = 3 in each control group and n = 4 in each PD group). Results were analyzed statistically by Two-Way ANOVA followed by Bonferroni test. Daily variation (17 and 17.5) was significantly different for p21, p27 and cyclin D1 (p<0.01). Interaction between age (17 and 17.5) and treatment was highly significant for phospho-p27 (p = 0.0071). *p<0.05 when compared to the respective control group. ^#^p<0.05 when compared to the same treatment at 17 d. Assays were conducted in triplicates.

In order to understand the mechanism by which ERK might influence gastric cell proliferation, we analyzed cell cycle-related proteins at 17 days in the morning (17 d) and evening (17.5 d), considering that their variation might precede the effects described for PI and MI. Also, both periods were checked because the circadian rhythm might affect protein concentration and cell cycle control. We evaluated p21, p27, cyclin E, CDK2, cyclin D1 and CDK4 by Western blot. The levels of the regulatory proteins did not change after PD98059 treatment in both periods of the day when compared to the respective control (DMSO) groups (**Figure 3A**). However, by checking the variation between 17 and 17.5 days amongst controls and treated groups, we observed higher levels of p21 and cyclin D1 in the evening (17.5) when compared to the samples collected in the morning (17) (p<0.05) (**Figure 3A**). Two-Way ANOVA confirmed that daily variation (17 and 17.5 d) is significantly different for both molecules (p<0.0001). These results suggested that both p21 and cyclin D1 were influenced by a circadian rhythm during early weaning, but such responses were not dependent on MEK (Figure 3A), since only cyclin D1 was disturbed by ERK inhibition.

When we checked for the circadian changes on p27, we found a significant decrease in the evening (p<0.05) (17.5) only in the control group (**Figure 3A**). Because p27 stability is responsive to ERK signaling [25], we also investigated p27 activation after ERK inhibition by studying the levels of p27 phosphorylated at threonin 187 (phospho-p27) in the gastric epithelium of 17- and 17.5-day-old early-weaned rats treated or not with PD98059. Amongst control groups, we observed increased concentrations of phospho-p27 in 17.5 -day-old animals in comparison to the 17-day-old group, suggesting that phosphorylation of p27 at threonin 187 presented a circadian variation (**Figure 3B**). By using Two-way ANOVA, we showed that age (17 and 17.5 d) and treatment interaction was highly significant (p = 0.0071), indicating that the effect of PD98059 depended on the period of the day. Additionally, we performed the Bonferroni posttest and we found that ERK inhibition decreased phospho-p27 levels in the gastric mucosa at 17.5 days when compared to the respective control group (p<0.05) (**Figure 3B**).

## Discussion

Gastric cell proliferation is regulated by a combination of endogenous and exogenous factors that generate pro- and anti-mitogenic signals, and thus, the imbalance between these signals results in stimulated or inhibited cell division. During postnatal development, the contribution of growth factors, diet, hormones and milk-borne molecules to the growth of the stomach is well described, but it is still unclear how these factors interact and which mechanisms are triggered to control gastric epithelial proliferation. The early-weaning model is suitable to study the importance of suckling during the postnatal development, because it promotes a change in the feeding pattern and the withdrawal of milk-borne molecules, such as hormones, antibodies and growth factors. This abrupt alteration leads to increased gastric cell proliferation [17] and differentiation [15], concomitant with higher levels of TGFα and its receptor EGFR [15]. Both molecules play multiple roles in the gastric cells *in vivo* and *in vitro*, including the stimulus to cell division and maturation [15], [20], [26], [27], inhibition of apoptosis [28], and augment of cell migration and regeneration [27], [29], [30]. The involvement of EGFR in the proliferative response promoted by early weaning has been demonstrated by inhibition of its phosphorylation, which resulted in a reduction of approximately 14% in the mitotic index [20]. However, between EGFR activation and the proliferative response itself there are unknown mechanisms triggered by the receptor to influence gastric cell proliferation during early weaning.

EGFR is able to activate several signaling pathways, and among them are MAPK/ERK, PI3K and Src cascades, which influence cellular kinetic processes [21]. Despite the plethora of effects, the phosphorylation of ERK and Src is high in the gastric mucosa of early-weaned rats [20], suggesting that they could be part of the gastric developmental responses towards feeding change. Interestingly, the inhibition of EGFR activation impairs ERK phosphorylation in the gastric mucosa during early weaning and decreases epithelial cell proliferation, whereas phosphorylation of Src remains unchanged [20]. These results point to MAPK as the main signaling pathway involved in the proliferative stimulus in the gastric mucosa of early-weaned animals, even though Src participation could not be totally excluded. Accordingly, TGFα induces AP-1 activation in the stomach of aging rats in ERK-dependent manner, with a minor participation of Src signaling pathway [31]. Also, the mechanical stimulation of gastric cells increases c-fos and c-myc expression and AP-1 activation, and stimulates cell proliferation through ERK [32]. Our results demonstrated that the inhibition of MAPK through PD98059 reduced ERK phosphorylation and did not alter p-Src levels, confirming that this tool could be used in vivo to modify MAPK activity.

We investigated whether ERK is part of the mechanism triggered by early weaning to stimulate cell proliferation in rat gastric mucosa. Our results showed that inhibition of ERK phosphorylation impaired epithelial proliferation in early-weaned animals. The reduction in mitotic and proliferative indexes was of 16–18% after PD98059 treatment, which is close to the decrease of 14% previously observed in early-weaned rats submitted to EGFR inhibition [20]. These results suggest that MAPK is activated by EGFR during early weaning leading to increased cell proliferation. Accordingly, *in vitro* studies with gastric and intestinal cells demonstrated that inhibition of ERK activation impairs the proliferative response triggered by diverse stimuli such as transmural pressure [32], fibroblast growth factor (FGF) [33], sonic hedgehog [34], leptin [35], EGF [36], apo-lactoferrin [37], [38] and glucagon-like peptide 2 [39]. In rats, gastric ischemia-reperfusion causes mucosal injury that is rapidly repaired, but this healing process is obstructed when PD98059 is administered to the animals and consequently the lesion is aggravated due to augmented apoptosis and decreased proliferation [40]. Inhibition of ERK activation also reduces the proliferation promoted by apo-lactoferrin, an iron-binding glycoprotein, in isolated mouse crypt cells [38]. After confirming the involvement of MAPK signaling pathway in cell proliferation induced by early weaning, we moved to the next question: how could ERK interfere with cell cycle control in this growth stimulatory condition?

Cyclins and CDKs play leading roles in cell cycle, but besides the formation of complexes, numerous other molecules interact to influence positive or negatively the regulatory mechanism. We found that the levels of cyclin D1, p21 and p27 changed when morning and evening samples were compared for control pups, indicating that these proteins normally vary in a circadian way. Such oscillation has been described for cell division in the gastric mucosa of adult rats that present peaks of proliferative indices in the morning [41], which could result from a variation of cell cycle-related proteins. In addition, we found increased levels of cyclin D1 and p21 concomitant with low concentration of p27 in DMSO-control group (17.5), which is consistent with the gastric proliferative stimulus reported for early-weaned 18-day-old rats [17]. Although p21 is mainly known as a cell cycle inhibitor, an opposite role has also been associated with this CKI [42]. Accordingly, inhibition of EGFR activity in early weaned rats leads to decreased gastric cell proliferation concomitantly with decreased levels of p21 [20]. Fasting is another feeding change that induces cell proliferation in the gastric mucosa of suckling rats [43], and it also augments p21 levels shortly after the withdrawal of food [44]. Therefore, the pro-proliferative function of this CKI [42] cannot be excluded from our results. When we evaluated the effect of ERK inhibition on cell cycle proteins, we observed that most of them were not altered by PD98059 treatment, suggesting that during early weaning other signaling pathways might get involved to control cyclin D1, p21 and p27.

As we mentioned, we also demonstrated that the concentration of p27 was not affected in the gastric mucosa of early-weaned rats after the inhibition of ERK phosphorylation. However, as post-translational events can modulate p27 activity, we investigated one of the steps involved in degradation [45]. It is known that cyclin E-CDK2 complex phosphorylates p27 at threonin 187, targeting the CKI for proteasome [10], [11]. After that, phospho-p27 is recognized by SKP2 [12], ubiquitylated and then degraded through UPS. We found that at 17.5 days, the inhibition of ERK significantly reduced p27 phosphorylation, indicating the lower degradation of this CKI after PD98059 treatment. Such results suggest that MAPK signaling pathway interferes in the regulation of p27 function in the gastric mucosa of early-weaned animals, possibly by controlling one of the steps involved in degradation process.

The role of MAPK on p27 has been reported *in vitro* for epithelial and fibroblastic cells. In response to serum stimulation, p27 is down-regulated in intestinal IEC-6 cells and CCL39 fibroblasts, and this effect is abolished or reduced by PD98059 [46], [47]. Adding to that, in NIH 3T3 asynchronous cells, the inhibition of MAPK activation leads to p27 accumulation as a consequence of lower degradation, but differently from our findings, this event is independent of phosphorylation at threonin 187 [47]. Conversely, the FGF2-induced proliferation of human corneal endothelial cells is accompanied by phosphorylation of p27 at serine 10 and threonin 187, and after MEK inhibition the phosphorylation at both sites is reduced [48]. More recently, Huang et al. [25] showed that cell proliferation is stimulated by estrogen in endometrial carcinoma and endometrial epithelial cells through ERK1/2-dependent phosphorylation of p27 at threonin 187 and its subsequent degradation.

In summary, we showed that the proliferative stimulus induced by early weaning in the rat gastric mucosa involves the activation of MAPK pathway that in turn, promotes the phosphorylation of p27 at threonin 187. Therefore, during early weaning, this cascade should allow cell cycle progression and contribute to the induction of epithelial cells proliferation. We suggest that this mechanism might be essential to regulate the rapid growth of gastric mucosa triggered by the abrupt transition from milk to solid food.

## Materials and Methods

### Ethics statement

All animal experimentation conducted during this study was approved by the Ethical Committee on Animal Use of the Institute of Biomedical Sciences of the University of Sao Paulo (CEUA protocol numbers 124/06 and 80/11). All animal manipulations were performed by trained personnel.

### Animals and early weaning

Wistar rats from the Animal Colony at the Department of Cell and Developmental Biology (ICB USP) were kept at 22°C and under a 12 h light:12 h dark cycle with water offered *ad libitum*. Pregnant females were kept in isolated cages and delivery was set as day 0. Litters were culled to 8–9 pups around the third postnatal day. In order to start early weaning, pups from different litters were separated from the dams at 15 days and placed in plastic cages with water and hydrated powdered chow (Nuvilab CR-1, Nuvital Nutrientes SA) *ad libitum*. Because they might not defecate and urinate due to the absence of the dam, these functions were stimulated by abdominal massage twice a day. Body weight was checked throughout the experiment.

### Administration of PD98059

PD98059 (Calbiochem) inhibits ERK phosphorylation by blocking MEK activity. In order to investigate the role of MAPK signaling pathway in gastric cell proliferation stimulated by early weaning, we administered PD98059 to rats under this dietary condition. After the onset of early weaning, pups were daily injected intraperitoneally with vehicle (0.5% DMSO) as control or PD98059 at 300 µg/kg. All animals received the last injection 30 min before euthanasia.

### Stomach collection

At least three animals from both control and PD98059-treated groups were euthanized at 17 and 18 days, which means two and three days after the onset of treatment. Sample collection occurred at 10h00 and 22h00 on day 17, and at 10h00 on the 18^th^ day. Pups were anesthetized with a 1∶1 (v/v) mixture of xilazyne (Anasedan, Vetbrands) and ketamine chloridrates (Dopalen, Vetbrands) (0.5 ml/100 g body weight). Stomachs were immediately excised, opened by the small curvature, flushed with 0.9% saline solution and submitted either to mucosa scraping or fixation in 10% formaldehyde.

### Western blot

Western blot was performed as previously described [20]. Briefly, gastric mucosa of the corpus region was scraped and immediately used for protein extraction with RIPA lysis buffer (150 mM NaCl, 1% NP-40, 1% sodium deoxycolate in 50 mM Tris-HCl, pH 7.5) containing protease and phosphatase inhibitors (1 mM PMSF, 0.45 mg/ml benzamidin, 1 mM leupeptin, 1 mM aprotinin, 5 mM sodium orthovanadate) (Sigma Chemical). Thirty µg of total protein were separated into 5–20% gradient SDS-PAGE and transferred to nitrocellulose membranes (Hybond-ECL, GE Healthcare). Samples were incubated overnight at 4°C with rabbit polyclonal antibodies to the signaling proteins ERK1 (1∶5000, sc-93, Santa Cruz Biotechnology) and phospho-Src (1∶300, #2101, Cell Signaling Technology), and to the cell cycle proteins cyclin E (1∶200, sc-481), CDK2 (1∶200, sc-163), cyclin D1 (1∶200, sc-718), CDK4 (1∶300, sc-601), p27 (1∶100, sc-528) (Santa Cruz Biotechnology), and phospho-p27 (T187, 1∶750, ab75908, ABCAM). Monoclonal antibodies were used to detect phospho-ERK (1∶5000, sc-7383, Santa Cruz Biotechnology), Src (1∶1000, #2110, Cell Signaling Technology) and β-actin (1∶10000, A5441, Sigma Chemical). Reactions were developed with ECL Kit (GE Healthcare) and signals were registered on X-ray films (MXG-Plus, Kodak). Densitometry was performed with Image J (1.37v Software, NIH Public Domain).

### Cell proliferation analyses

Non-serial 6 µm paraffin sections were submitted either to immunohistochemistry for PCNA or to staining with Hematoxylin and Eosin (HE), which were respectively used to determine proliferative (PI) and mitotic indices (MI).

For PCNA immunohistochemistry, sections were rehydrated with 0.05 M phosphate buffered saline (PBS), the endogenous peroxidase was blocked with 0.3% H_2_O_2_ in methanol (30 min) and antigen retrieval was achieved by boiling slides in citric acid (pH 6). Non-specific binding was blocked by 20% goat serum (30 min). Tissue sections were incubated overnight at 4°C with monoclonal antibody for PCNA (1∶50, M0879, DAKO). After washing, a peroxidase-conjugated anti-mouse antibody (1∶50, Jackson ImmunoResearch Laboratories) was added, and the reaction was developed by 0.05% 3,3′- diaminobenzidine (DAB) (DAKO) in PBS containing 0.15% H_2_O_2_. Slides were counterstained in Mayer's Hematoxylin and negative controls were incubated with normal serum to replace the primary antibody.

The MI was determined in HE stained histological sections. Both PI and MI were obtained under light microscope (Nikon) at×800 magnification using an integrative eyepiece with ocular grid (Zeiss, Germany). We estimated PI by counting around 2,500 epithelial cells as PCNA-immunolabeled or nonlabeled along the proliferative compartment. Only longitudinal sections were considered. PI was determined for each animal as PCNA-labeled cells/total epithelial cells×100. MI was determined in HE stained histological sections in which mitotic and interphasic cells were counted following the same criteria above. MI was represented as mitotic cells/total epithelial cells×100.

### Statistical analyses

Results were analyzed by Student *t*-test or Two-Way ANOVA followed by Bonferroni test (GraphPad Prism 5.01, GraphPad Software, Inc.) when applicable to evaluate differences between groups. Significance level was set at p<0.05.
